# Use of the Archimedes navigation system to diagnose peripheral pulmonary lesions: preliminary Italian results

**DOI:** 10.3389/fonc.2024.1394022

**Published:** 2024-05-15

**Authors:** Filippo Lanfranchi, Laura Mancino, Gabriele Foltran, Lorenzo Nicole’, Lucio Michieletto

**Affiliations:** ^1^ Respiratory Disease Unit, Department of Cardiac Thiraciuc and Vascular Sciences, Ospedale dell’Angelo, Venice, Italy; ^2^ Pathology Unit, Ospedale dell’Angelo, Venice, Italy

**Keywords:** bronchoscopy, lung cancer, peripheral pulmonary lesion (PPL), virtual bronchoscopy navigation (VBN), bronchoscopic transparenchymal nodule access (BTPNA)

## Abstract

Diagnosis of peripheral pulmonary lesions (PPL) is one of the most challenging fields in early lung cancer diagnosis. Despite novel techniques and new approaches to the periphery of the lung, almost 25% of PPL remains undiagnosed. Virtual bronchoscopy navigation (VBN) potentially allows to sample PPL previously not reachable with conventional bronchoscopy. In this preliminary report, we described nine cases of PPL (in which conventional bronchoscopy did not reach the lesion) sampled with VBN, from which we obtained a diagnosis in seven out of nine cases (77.8%), consistent with other reported results in literature. More large-scale data are needed to whether VBN can increase diagnostic yield (DY) of PPL.

## 1 Introduction

Lung cancer is the most fatal and the second most common cancer worldwide. A lung nodule, or lesion, can represent the earliest detectable stage of lung cancer. It has been well demonstrated that the stage of diagnosis is inversely related to prognosis, with early detection leading to significant improvements in survival (1). Obviously, as the number of patients with lung nodules increases, there will be increased demand to perform tissue sampling.

Traditionally, peripheral pulmonary lesion (PPL) is considered one of the most challenging fields in bronchoscopy. Despite novel techniques and new approaches to the periphery of the lung, almost 25% of PPL remains undiagnosed.

Over time, novel endoscopic approaches have been developed to sample PPL: transbronchial lung biopsy (TBLB) with fluoroscopy, radial probe endobronchial ultrasound (R-EBUS), electromagnetic navigational bronchoscopy (ENB), cone-beam CT (CBCT)-assisted bronchoscopy, and robotic bronchoscopy.

VBN is an emerging tool for PPL diagnosis, allowing to navigate close to PPL otherwise not reachable with conventional bronchoscopy.

In one randomized controlled trial (RCT), the authors evaluated the value of VBN-assisted bronchoscopy (VBN in conjunction with the ultrathin bronchoscope and fluoroscopy) and did not find a significant higher DY in the VBN group compared with the non VBN-group (67.1% vs. 59.9%, p = 0.173) (2). Another RCT evaluated the added value of VBN in conjunction with fluoroscopy and R-EBUS and showed a significant higher DY in the VBN group compared with non-VBN group (R-EBUS and fluoroscopy alone) (80.4% vs. 67.0%, p = 0.032) (3).

The difference between these results could be due to the bronchial anatomy: in fact, if the lesion is not reached by a bronchus (absence of bronchus sign), the likelihood of obtaining an accurate diagnosis through transbronchial biopsy diminishes.

To overcome this limitation, bronchoscopic transparenchymal nodule access (BTPNA) approaches have been developed to create a pathway toward the lesion. This technique consists of creating a hole in the airway wall at the point of entry with a needle and then carrying out sampling at this level, thus allowing nodules without a bronchus sign to be reached with bronchoscopic instruments.

The Archimedes System (Archimedes™ System Broncus Medical^©^, San Jose, CA) is an image-guided bronchoscopy navigation system that uses the VBN approach and BTPNA.

**Figure 1:**
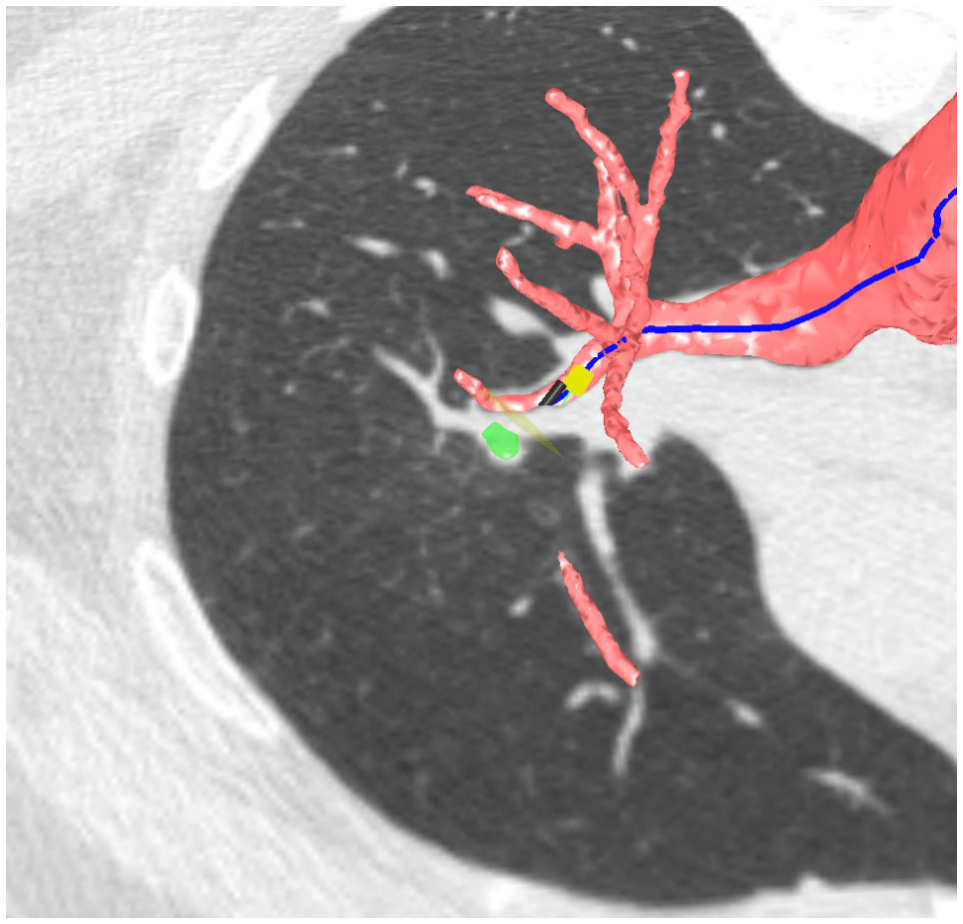
Archimedes’ 3D pathway reconstruction.

The Archimedes System reconstructs chest CT images into a 3D model and allows to mark lesions to be sampled. The procedures are planned with a preoperative CT scan, which is imported into the Archimedes system, and a 3D reconstruction of the airway and vessels is automatically generated (**Figure 1**). The possible pathways are calculated for the lesion (up to eight different lesions). A point-of-entry (POE) location and path are planned, and the procedure plan is exported. On the day of the procedure, the patient was placed on the same dedicated bed and a C-arm was used to perform fused fluoroscopy during VBN, in order to synchronize the patient’s position and the lesion marked at chest CT scan with fluoroscopic images. This can be possible by using two electromagnetic plates: one was placed on the C-arm and the second one on the patient’s bed. This technique allows to reach lesions without bronchus sign, following the pathway provided by Archimedes’ system, performing a BTPNA, which is one of the strengths of this VBN system.

The first study performed in humans was made by Herth and colleagues in 2015 (4). In 10/12 patients (82%), the procedure was successfully performed with adequate tissue sampling. The most recent study of Sun performed in 114 patients revealed a BTPNA DY of 93.9% and sample adequacy for definite diagnosis in 75.4% of the cases (5).

Here, we present our preliminary results by using Archimedes System VBN, wherein we sampled nine cases of PPL. These lesions had been previously sampled using bronchoscopy and R-EBUS with inconclusive results.

## 2 Material and methods

Between April and June 2023, nine patients underwent VBN with biopsies in our Pulmonology Unit (Ospedale dell’Angelo, ULSS3 Serenissima, Venice-Mestre, Italy). All the patients presented PPL on chest CT scan. All patients underwent a conventional bronchoscopy with R-EBUS, fluoroscopy, and TBLB, and diagnoses were not obtained. A written consent was obtained from all patients.

Procedures were performed under deep sedation at the discretion of the anesthesiologist and with a laryngeal mask. A VBN with fused fluoroscopy (Archimedes™ System Broncus Medical^©^, San Jose, CA) was used.

**Figure 2:**
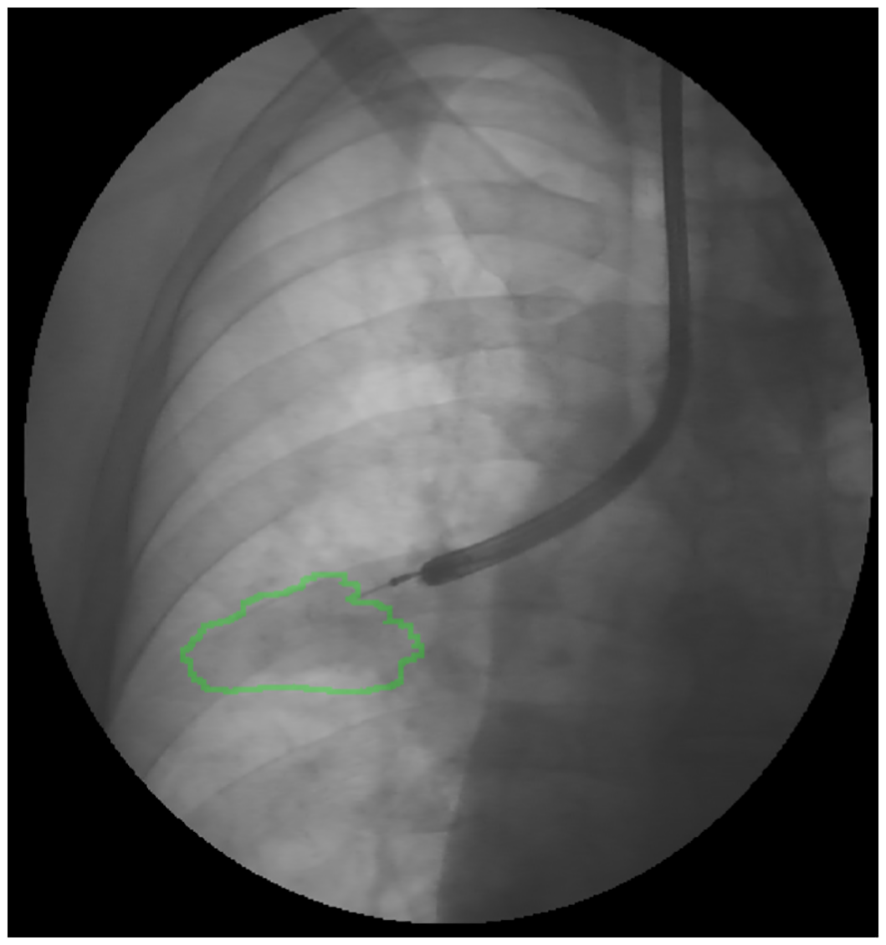
Fused fluoroscopy: localization of the lesion during bronchoscopy with R-EBUS.

**Figure 3:**
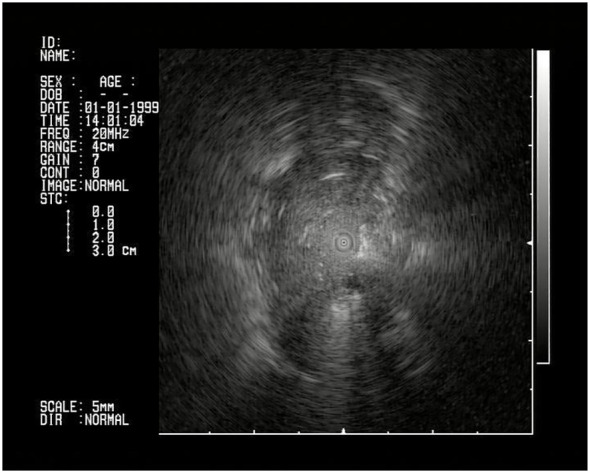
R-EBUS confirmation of the lesion.

The procedures were planned as follows: the day before the exam, a preoperative CT scan was imported into the Archimedes system and a 3D reconstruction of the airway and vessels was automatically generated (**Figure 1**). A point-of-entry (POE) location and path were planned, and the procedure plan was exported onto the Archimedes’ system. Biopsies were performed by using bronchoscopes (Fujifilm 580T, Fujifilm, Tokyo, Japan, outer diameter 5.8 mm, working channel 2.8 mm) with forceps (2.0-mm forceps, Olympus, Tokyo, Japan); localization of the lesion and confirmation of the right site of biopsy were done in all cases with R-EBUS and fused fluoroscopy (**Figures 2**, **3**).

**Figure 4:**
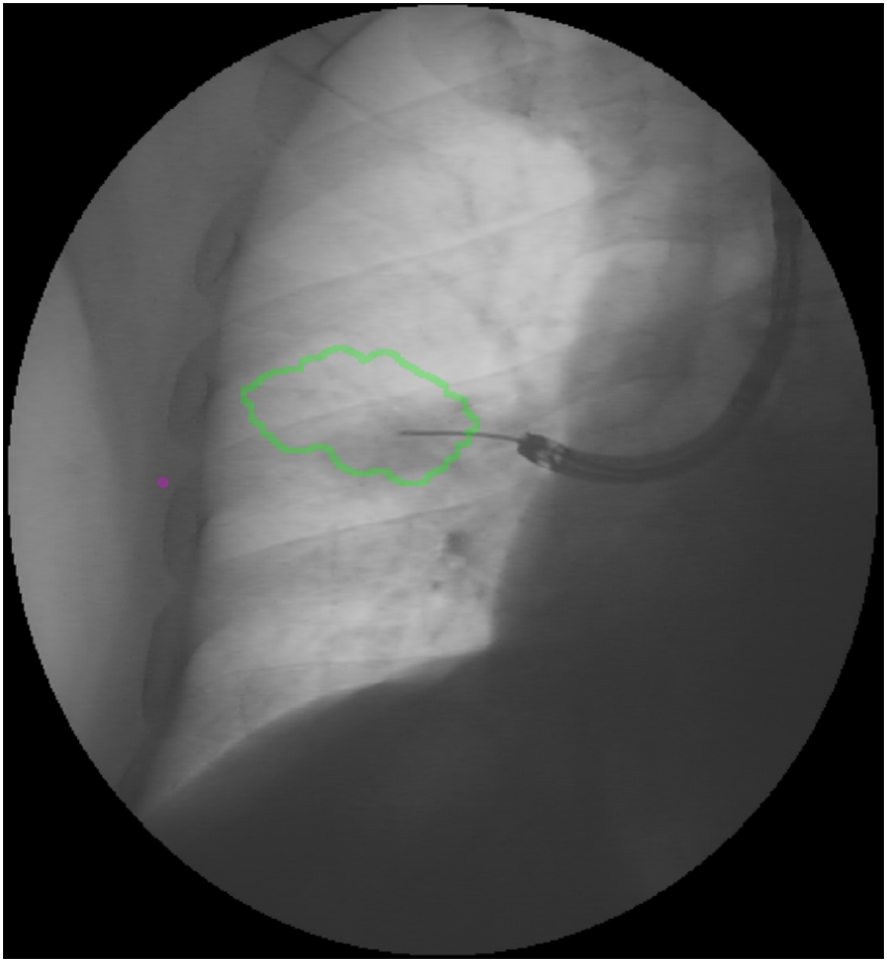
Fused fluoroscopy: BTPNA sampling.

**Figure 5:**
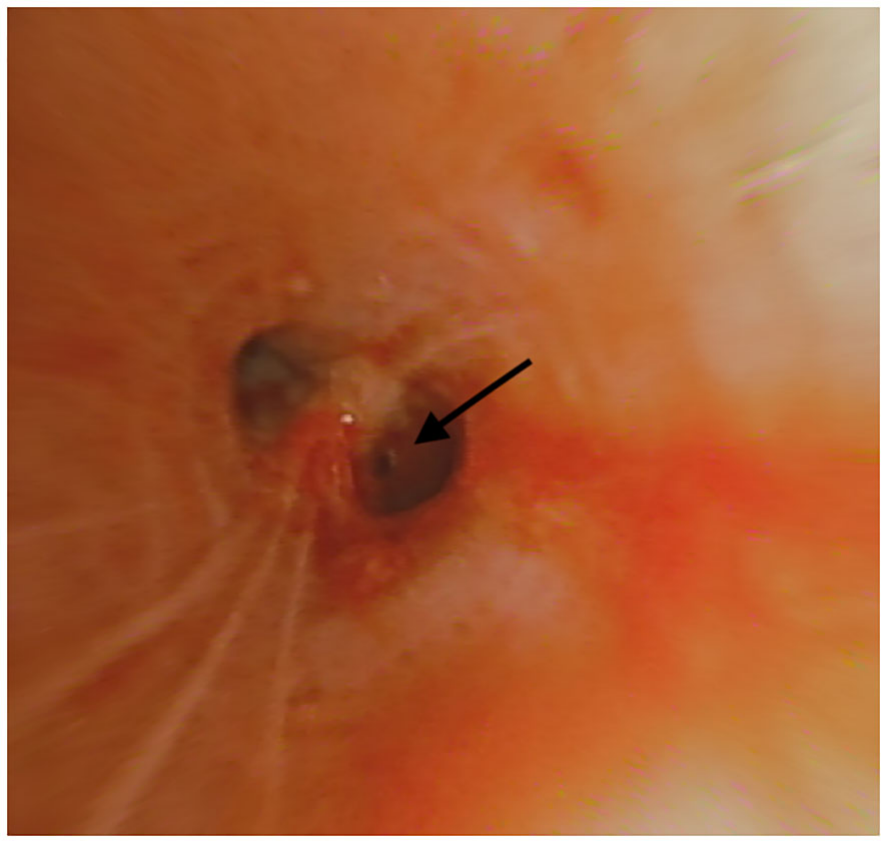
Point-of-entry for BTPNA (right arrow).

In cases in which bronchus sign was not present, a BTPNA was performed: a POE was created at the location using the tools contained in the Archimedes Access Kit (Broncus Medical^©^, San Jose, CA): an 18-gauge needle (FleXNeedle^®^, Broncus Medical^©^, San Jose, CA), a dilatation balloon, and a sheath. Once the POE location was reached, the needle was inserted through the working channel of the bronchoscope, followed by a balloon dilation. A sheath was inserted through the hole and advanced until the lesion with a blunt tipped stylet toward the lesion under fused CT/fluoroscopic guidance, and biopsies with miniforceps (CoreDx miniforceps, Boston Scientific, Watertown, MA) when feasible or transbronchial needle aspiration (TBNA) were performed (**Figures 4**, **5**). Rapid on-site evaluation (ROSE) was not used.

A sample was considered positive if malignant cells were present; DY was defined as the number of sample-positive patients divided by the total cohort size. All samples were evaluated by the Pathology Unit of the same Hospital (Ospedale dell’Angelo, ULSS3 Serenissima, Venice-Mestre, Italy). Complications were also recorded, in terms of bleeding, pneumothorax/pneumomediastinum, respiratory failure, cardiac failure, hemodynamic instability, and death.

## 3 Results

Patients’ and peripheral pulmonary lesions’ characteristics, tools used, and diagnosis are shown in **Table 1**.

**Table 1:** Patients’ and peripheral pulmonary lesions’ (PPL) characteristics.

Patient N	Sex (m/f)	Age	Lesion size (mm)	Position	Bronchus sign yes/no	TBLB yes/no	TBNA yes/no	BTPNA yes/no	Diagnosis
N.1	F	60	21	RB2	Y	Y	Y	N	NOS k
N.2	M	76	41	RB2	N	N	Y	N	Nd
N.3	M	53	13.8	RB1	Y	Y	Y	N	CCRM
N.4	M	41	22.4	RB6	Y	Y	Y	N	Adk
N.5	M	75	27.2	LB3	N	Y	Y	N	NOS k
N.6	M	52	20.2	RB3	N	N	Y	Y	SCC
N.7	M	82	17.5	LB4	N	N	N	Y	BrCAM
N.8	M	72	37.4	RB2	Y	Y	N	N	Adk
N.9	M	82	27	RB4	N	N	N	Y	Nd

NOS k: carcinoma not otherwise specified. Nd, non-diagnostic; CCRM, colorectal cancer metastasis; Adk, adenocarcinoma; SCC, squamous cell carcinoma; BrCAM, breast cancer metastasis.

The mean size of the lesions was 25.3 mm (13.8–41). All of the lesions were solid, no ground glass opacities (GGO) were sampled.

Of the nine patients who underwent VBN, seven of them (77.8%) received a diagnosis. Among the two cases with non-diagnostic results, one underwent a surgical lung biopsy (SLB), revealing a urothelial cancer metastasis. The second patient underwent a CT-guided chest trans thoracic needle biopsy (CT-TTNB), which revealed a squamous cell carcinoma (SCC).

In five cases of nine, sampling and diagnosis were established by using conventional forceps, whereas in three of them a BTPNA approach was used: the latter were sampled with a TBNA or with miniforceps approach.

In BTPNA approaches, in one case both TBNA and biopsies were performed and yielded positive results (for squamous cell carcinoma—SCC). In another case, only biopsy (with miniforceps) was performed and was positive (for breast cancer metastasis—BrCAM). In the last case which was non-diagnostic, only biopsy was performed.

Regarding complications, in case 2, hemodynamic instability occurred allowing only a single TBNA sample to be performed. In case 7, a post-procedure chest X-ray (CXR) revealed pneumomediastinum without any clinical significance (no oxygen desaturation or hemodynamic instability). The patient was discharged 5 days after the procedure without complications. No bleeding, respiratory or cardiac failure, or death occurred.

## 4 Discussion

This study provides an initial assessment of the utilization of a bronchoscopic navigational system in Italy, where its usage is not yet widespread. In this preliminary report, we assessed the Archimedes VBN approach in nine patients with PPL for whom conventional bronchoscopy did not yield a final diagnosis, achieving a DY of 77.8%.

The diagnostic yield of various bronchoscopy modalities has been extensively researched. In the current literature, recent meta-analyses have shown an overall DY for conventional bronchoscopy with R-EBUS (with or without a guide sheath) around 72% (6). Actually, whatever navigational technique used, DY now rarely exceeds 75% (7–9) with the lack of needle in target lesion confirmation (i.e., “Tool in lesion”) as the key limiting factor. Thus, novel techniques regarding needle imaging might be helpful tools to identify malignant lesions and confirm the right place for biopsy, enabling optimal tissue acquisition (10). Our findings suggest that, in selected cases where conventional bronchoscopy fails to yield a diagnosis, VBN with or without BTPNA may be considered to enhance the overall DY.

Herth and colleagues in 2015 first described the use of the Archimedes system with BTPNA in diagnosis of lung lesion, reporting a DY of 82% (4). In 2022, Sun et al. conducted a prospective, single-arm, multicenter study in which they evaluated the use of the Archimedes system on 104 patients with lung lesion, reporting a DY (after follow-up) of 72.8%–75.4% (5). Hiddinga et al. in a single-center, prospective, observational cohort study reported a DY of 77% in 35 patients who underwent the virtual bronchoscopy navigation procedure (11).

Overall, considering the variations in study designs, our data align with those previously described. In addition, compared with these studies, our study involved patients who previously underwent bronchoscopy with the aid of R-EBUS without receiving a definitive diagnosis.

In summary, despite requiring specific training and more time than conventional bronchoscopy in terms of preprocedural planning (chest CT scan, navigational planning, need for a deep sedation, room setup, time of procedure), our data suggest that VBN may help reach a diagnosis even in those patients in whom conventional bronchoscopy had not been sufficient.

Regarding complications, we reported a complication rate of 22% (2/7), including one case of hemodynamic instability (maybe linked to the anesthesia) and one case of pneumomediastinum. The pneumomediastinum was likely associated with the BTPNA procedure. These data also appear in line with those reported in other studies that used Archimedes.

This study has several limitations, starting from the number of patients and the absence of standardization in sampling: further studies are needed to confirm and ascertain whether VBN can be considered for peripheral lesions in cases where conventional bronchoscopy has failed to provide diagnostic results.

## 5 Conclusions

The field of interventional pulmonology for PPL analysis and treatment is evolving rapidly. A desirable future concept is the one-step bronchoscopic approach including navigation to the tumor, biopsy sample, and diagnosis for future treatments. PPL are the current challenge: several bronchoscopic guidance technologies have been developed that resulted in an improved diagnostic yield of peripheral lung lesions but data are still poor. In summary, our study provides an initial assessment of bronchoscopic navigational systems in Italy, indicating potential utility in cases where conventional bronchoscopy fails to diagnose peripheral pulmonary lesions. However, the data on these techniques are preliminary and further research is needed.

In conclusion, novel tools like VBN should be considered in pulmonology centers with high technology, as a useful tool to increase DY for PPL and have to be used as an adjunctive and not a substitute of conventional bronchoscopy, to raise DY as much as possible.

## Data availability statement

The raw data supporting the conclusions of this article will be made available by the authors, without undue reservation.

## Ethics statement

Ethical approval was not required for the studies involving humans because of the nature of the retrospective analysis. The studies were conducted in accordance with the local legislation and institutional requirements. The participants provided their written informed consent to participate in this study.

## Author contributions

FL: Conceptualization, Investigation, Writing – original draft, Writing – review & editing. LM: Investigation, Writing – review & editing. GF: Data curation, Writing – review & editing. LN: Data curation, Investigation, Writing – review & editing. LM: Supervision, Writing – review & editing.

## References

[1] CDC. NPCR and SEER—U.S. Cancer Statistics: Public Use Database . Available online at: www.cdc.gov/uscs.

[2] Asano F Shinagawa N Ishida T Shindoh J Anzai M Tsuzuku A. Virtual bronchoscopic navigation combined with ultrathin bronchoscopy. A randomized clinical trial. Am J Respir Crit Care Med. (2013) 188:327–33. doi: 10.1164/rccm.201211-2104OC 23600452

[3] Ishida T Asano F Yamazaki K Shinagawa N Oizumi S Moriya H. Virtual bronchoscopic navigation combined with endobronchial ultrasound to diagnose small peripheral pulmonary lesions: a randomised trial. Thorax. (2011) 66:1072–7. doi: 10.1136/thx.2010.145490 PMC3221323 21749984

[4] Herth FJ Eberhardt R Sterman D Silvestri GA Hoffmann H Shah PL. Bronchoscopic transparenchymal nodule access (BTPNA): first in human trial of a novel procedure for sampling solitary pulmonary nodules. Thorax. (2015) 70:326–32. doi: 10.1136/thoraxjnl-2014-206211 25746631

[5] Sun J Criner GJ Dibardino D Li S Nader D Lam B. Efficacy and safety of virtual bronchoscopic navigation with fused fluoroscopy and vessel mapping for access of pulmonary lesions. Respirology. (2022) 27:357–365. doi: 10.1111/resp.14224 35212090

[6] Sainz Zuñiga PV Vakil E Molina S Bassett RL Jr Ost DE. Sensitivity of radial endobronchial ultrasound-guided bronchoscopy for lung cancer in patients with peripheral pulmonary lesions: an updated meta-analysis. Chest. (2020) 157:994–1011. doi: 10.1016/j.chest.2019.10.042 31738928

[7] Furuse H Matsumoto Y Nakai T Tanaka M Nishimatsu K Uchimura K. Diagnostic efficacy of cryobiopsy for peripheral pulmonary lesions: A propensity score analysis. Lung Cancer. (2023) 178:220–8. doi: 10.1016/j.lungcan.2023.02.022 36893563

[8] Liu Y Wang F Zhang Q Tong Z. Diagnostic yield of virtual bronchoscope navigation combined with radial endobronchial ultrasound guided transbronchial cryo-biopsy for peripheral pulmonary nodules: a prospective, randomized, controlled trial. Ann Transl Med. (2022) 10:443. doi: 10.21037/atm 35571447 PMC9096374

[9] Chen AC Pastis NJ Mahajan AK Khandhar SJ Simoff MJ Machuzak MS. Robotic bronchoscopy for peripheral pulmonary lesions: A multicenter pilot and feasibility study (BENEFIT). Chest. (2021) 159:845–52. doi: 10.1016/j.chest.2020.08.2047 PMC7856527 32822675

[10] Kalchiem-Dekel O.r. Connolly JG Lin I-H Husta BC Adusumilli PS Beattie JA. Shape-sensing robotic-assisted bronchoscopy in the diagnosis of pulmonary parenchymal lesions. Chest. (2021). doi: 10.1016/j.chest.2021.07.2169 PMC8941601 34384789

[11] Hiddinga BI Slebos DJ David Koster T Hijmering-Kappelle LBM Hiltermann TJN Kievit H. The additional diagnostic value of virtual bronchoscopy navigation in patients with pulmonary nodules - The NAVIGATOR study. Lung Cancer. (2023) 177:37–43. doi: 10.1016/j.lungcan.2023.01.012 36708592

